# Reflection impulsivity in binge drinking: behavioural and volumetric correlates

**DOI:** 10.1111/adb.12227

**Published:** 2015-02-11

**Authors:** Paula Banca, Iris Lange, Yulia Worbe, Nicholas A. Howell, Michael Irvine, Neil A. Harrison, Michael Moutoussis, Valerie Voon

**Affiliations:** ^1^ Department of PsychiatryUniversity of CambridgeUK; ^2^ PhD Programme in Experimental Biology and BiomedicineCenter for Neuroscience and Cell BiologyUniversity of CoimbraPortugal; ^3^ Institute for Biomedical Imaging and Life SciencesFaculty of MedicineUniversity of CoimbraPortugal; ^4^ Behavioural and Clinical Neurosciences InstituteUniversity of CambridgeUK; ^5^ Cambridgeshire and Peterborough NHS Foundation TrustUK; ^6^ Brighton and Sussex Medical SchoolUniversity of SussexUK; ^7^ The Wellcome Trust Centre for NeuroimagingInstitute of NeurologyUniversity College LondonUK

**Keywords:** Binge drinking, decision making, reflection impulsivity, voxel‐based morphometry

## Abstract

The degree to which an individual accumulates evidence prior to making a decision, also known as reflection impulsivity, can be affected in psychiatric disorders. Here, we study decisional impulsivity in binge drinkers, a group at elevated risk for developing alcohol use disorders, comparing two tasks assessing reflection impulsivity and a delay discounting task, hypothesizing impairments in both subtypes of impulsivity. We also assess volumetric correlates of reflection impulsivity focusing on regions previously implicated in functional magnetic resonance imaging studies. Sixty binge drinkers and healthy volunteers were tested using two different information‐gathering paradigms: the beads task and the Information Sampling Task (IST). The beads task was analysed using a behavioural approach and a Bayesian model of decision making. Delay discounting was assessed using the Monetary Choice Questionnaire. Regression analyses of primary outcomes were conducted with voxel‐based morphometry analyses. Binge drinkers sought less evidence prior to decision in the beads task compared with healthy volunteers in both the behavioural and computational modelling analysis. There were no group differences in the IST or delay discounting task. Greater impulsivity as indexed by lower evidence accumulation in the beads task was associated with smaller dorsolateral prefrontal cortex and inferior parietal volumes. In contrast, greater impulsivity as indexed by lower evidence accumulation in the IST was associated with greater dorsal cingulate and precuneus volumes. Binge drinking is characterized by impaired reflection impulsivity suggesting a deficit in deciding on the basis of future outcomes that are more difficult to represent. These findings emphasize the role of possible therapeutic interventions targeting decision‐making deficits.

## Introduction

Binge drinking, a behaviour characterized by heavy ethanol intoxication followed by intermittent withdrawals, is a serious public health problem across countries, highly common in youths and young adults (Grucza, Norberg & Bierut 2009; Hibell *et al*. 2012; Johnston *et al*. 2014). This behaviour has been linked to several adverse health, social and economic consequences (Miller *et al*. 2007) and to an enhanced risk for the later development of alcohol‐use disorders (AUD; Crabbe, Harris & Koob 2011). The neurobiological effects of binge drinking include cognitive impairments in the domains of attention, working memory and executive function (Weissenborn & Duka 2003; Townshend & Duka 2005; Parada *et al*. 2012). Volumetric alterations have been found in binge drinkers, particularly in cerebellar and frontal cortices (McQueeny *et al*. 2009; Squeglia *et al*. 2012; Lisdahl *et al*. 2013). We have also previously shown that college‐age binge drinkers have greater ventral striatal volume relative to healthy volunteers. This may be related to either neuroplastic adaptation from repeated bingeing or withdrawal episodes or to a predisposing risk factor (Howell *et al*. 2013).

Converging evidence implicates heightened impulsivity underlying behavioural and substance‐use disorders as both state‐ and trait‐related factors (Perry & Carroll 2008; Robbins *et al*. 2012). Impulsivity can be divided into decisional and motor subtypes. Decisional impulsivity includes reflection impulsivity (the amount of information gathered before taking a decision) and delay discounting (a measure of the subjective discounting of a delayed reward). Motor impulsivity includes reduced motor response inhibition and premature or anticipatory responding (Voon *et al*. 2014).

A dysfunctional preference for immediate versus delayed reward has been consistently demonstrated in more severe forms of AUD (Bickel *et al*. 2014). The ventral striatal brain response on reward anticipation observed in AUD individuals was found to be inversely associated with impulsiveness as measured by means of Barrett's impulsiveness scale (Beck *et al*. 2009). The evidence for increased reflection impulsivity in AUD is less clear. Abstinent AUD individuals were not impaired on the matching familiar figures task (Weijers, Wiesbeck & Böning 2001) but made decisions at higher levels of uncertainty on the Information Sampling Task (IST), with a greater number of errors (Lawrence *et al*. 2009).

In rodents, repeated intermittent ethanol exposure during adolescence is shown to disrupt waiting and choice impulsivity and attention abilities (Sanchez‐Roige *et al*. 2014b). These findings have been recently translated in human young adult binge drinkers (Sanchez‐Roige *et al*. 2014a). Self‐reported impulsivity based on questionnaires has also been found elevated in binge drinkers (Stautz & Cooper 2013; Whelan *et al*. 2014). Specifically, negative‐urgency and sensation‐seeking traits appear to better predict heavy drinking (Stojek *et al*. 2014). Enhanced trait impulsivity is generally considered a vulnerability marker for substance‐use disorders (Verdejo‐Garcia, Lawrence & Clark 2008). Findings related to delay discounting in binge drinking are less consistent. Two studies that assessed adolescents with greater alcohol consumption (Vuchinich & Simpson 1998; Moreno *et al*. 2012), although not specifically defined as binge drinkers, reported elevated delay discounting. A third study (Whelan *et al*. 2014) also found hightened delay discounting in young adolescents binge drinkers (age 14) using the Monetary Choice Questionnaire, a result which was not predictive of future binge drinkers. However, a recent study found no differences between binge and non‐binge drinkers for this measure (Sanchez‐Roige *et al*. 2014a).

The present study focuses on the domain of decisional impulsivity, specifically reflection impulsivity. To our knowledge, only one study has assessed reflection impulsivity in binge drinking (Townshend *et al*. 2014). By using the IST, a paradigm that asks participants to decide which colour is predominant in a 5 × 5 matrix by opening boxes to make a decision, this study reported impairments in the ability to gather and evaluate information during decision making in high‐ compared with low‐binge drinkers. Here, we extend this study using not only the IST but also the beads task (both predominantly used to assess reflection impulsivity) to further assess its volumetric correlates. In the beads task, based on sequential viewing of coloured beads, subjects must decide from which jar the beads are being selected. This task has been applied to schizophrenia and behavioural and substance addictions including pathological gambling, illicit‐drug users and Parkinson's disease patients with behavioural addictions with high sensitivity to group differences (Djamshidian *et al*. 2012). We also assessed delay discounting using the Monetary Choice Questionnaire. We hypothesized that binge drinkers would have impairments in both forms of decisional impulsivity.

The accumulation of evidence has been shown to implicate regions including the parietal cortex, insula and dorsolateral prefrontal cortex (DLPFC) in studies involving single‐unit recordings and functional magnetic resonance imaging (fMRI; Shadlen & Newsome 2001; Basten *et al*. 2010; Stern *et al*. 2010; Furl & Averbeck 2011). An fMRI study in healthy volunteers (HV) using the same task used here (the beads task) found that higher evidence seeking was associated with greater parietal responses. The authors suggest that parietal cortex is specifically involved in comparing the costs of seeking additional evidence to the potential gains and losses of an immediate reward‐related decision. Additionally, insula responses were modulated by the action values or expected reward, which highlighted the insula's role in weighing the value of the decision (Furl & Averbeck 2011). Previous studies have also linked insula to the processing of uncertainty, risk and task difficulty (Heekeren *et al*. 2004; Huettel, Song & McCarthy 2005). The DLPFC has been suggested to be responsible for integrating and weighing costs against benefits by combining neural benefit and cost signals from the ventral striatum and amygdala, respectively (Basten *et al*. 2010). In the second component of the study, we examined the volumetric neural correlates of the two differing measures of reflection impulsivity investigating the relationship with brain volume using voxel‐based morphometry (VBM). We focused on the aforementioned regions hypothesizing a relationship between greater impulsivity and decreases in brain volume in these regions.

## Methods

### Recruitment

Binge‐drinking subjects (BD) were recruited by local advertisements in both community‐ and university‐based settings in the East Anglia region and the HV were recruited from the Behavioural and Clinical Neurosciences Institute healthy volunteer list. Binge drinking was assessed using the diagnostic criteria for binge drinking from the National Institute on Alcoholism and Alcohol Abuse (NIAAA 2004): consumption of ≥ 5 drinks and ≥ 4 drinks in a 2‐hour period (for males and females, respectively) at least once a week for the last 3 months. The BD also had to indicate that their drinking was motivated by a desire to get drunk and reported intoxication with each binge‐drinking episode.

All participants were greater than 18 years old, had no other substance‐use disorders and were free from any head injury or major neurological, medical or psychiatric disorders [as screened with the MINI International Neuropsychiatric Inventory (Sheehan *et al*. 1998)]. Participants were asked to refrain from alcohol consumption at least 24 hours before the experiments and were excluded if they had positive urine drug screen or alcohol breathalyzer test on the day of testing.

Subjects completed the AUD Identification Test (AUDIT) (Saunders *et al*. 1993), Beck Depression Inventory (Beck *et al*. 1961), the Spielberger State and Trait Anxiety Inventory (Spielberger 1985). Trait impulsivity was measured by the UPPS‐P Impulsive Behaviour Scale (Whiteside & Lynam 2001). Impulsive choice was assessed using the Monetary Choice Questionnaire (Kirby & Marakovic 1996) and reflection impulsivity was assessed using the IST and beads task. Sixty subjects completed the behavioural study. Twenty‐one subjects across both groups returned for the MRI scan with eight additional subjects recruited for the MRI study. The study was approved by the University of Cambridge Research Ethics Committee and written informed consent was obtained. Subjects were reimbursed for their participation and travel and received an additional amount dependent on performance.

### Beads task

**Figure 1:**
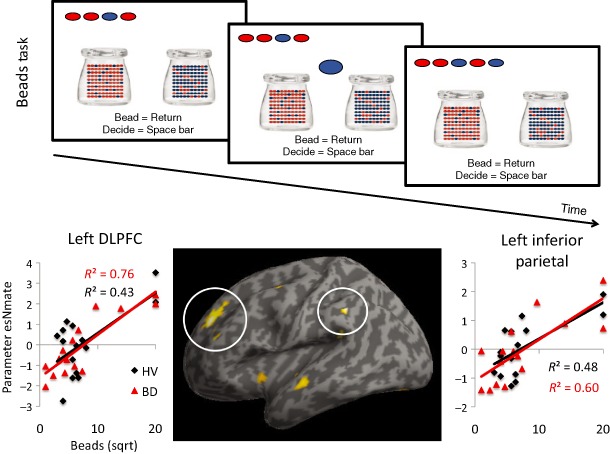
Beads task and relationship with brain volumes. Top panel: Beads task. Subjects viewed two jars with opposite ratios of red and blue beads (Jar 1: *P* = 0.80 red; *P* = 0.20 blue/Jar 2: *P* = 0.80 blue; *P* = 0.20 red). Beads selected from a single jar were sequentially shown to the participants. The goal was to infer from which jar the beads were being selected. After each bead was drawn, participants either chose to draw another bead or to make a decision. The drawn beads remained on display at the top of the screen. Bottom panel: Left dorsolateral prefrontal (DLPFC) and left inferior parietal cortices volumes positively correlated with the number of beads drawn in beads task. The data are shown for healthy volunteers (HV) and binge drinkers (BD)

Subjects were shown two jars on the computer screen with opposite ratios of red and blue beads (Jar 1: *P* = 0.80 red; *P* = 0.20 blue/Jar 2: *P* = 0.80 blue; *P* = 0.20 red) (Fig. 1). They were informed of the bead ratio and were told that beads from one of the jars would be presented one at a time in the centre of the screen. The subjects' goal was to infer whether the beads were drawn from Jar 1 or Jar 2. The subjects were free to view as many beads as they wanted to a maximum of 20 beads before committing to their decision. The decision was followed by a confidence rating in which subjects used a mouse to indicate the degree of confidence that their answer was correct on a line anchored at ‘Not confident’ to ‘Very confident’. Subjects were then informed that the next block would start. In this version, there was no feedback. The task controlled for working memory by showing the coloured beads drawn across two rows at the top of the screen. There was no time limit to the task. The primary outcome measure was the number of beads drawn prior to a decision. There were three blocks of trials with the same bead order used in a previous study (Moutoussis *et al*. 2011).

Following the MRI, subjects were tested on a modified version of the beads task with feedback in which subjects were told they would receive £1 for each correct decision. This version of the beads task with reward feedback was used as a covariate with the MRI data as more subject data were available. Data from 29 subjects (BD *n* = 14; HV *n* = 15) were used for the correlation analysis.

### IST

**Figure 2:**
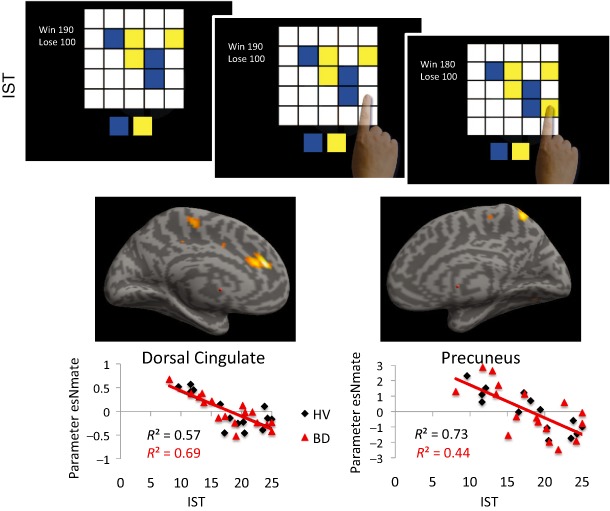
Information Sampling Task (IST) and relationship with brain volumes. Top panel: IST. A 5 × 5 matrix of grey boxes were presented on a touch‐screen monitor. Upon being touched, each box opened to reveal one of two colours, shown as panels below the matrix. The goal was to decide which colour was predominant in the matrix. The ‘fixed win’ condition is shown in which the number of points decreased by 10 points for every box touched. Bottom panel: Left dorsal cingulate cortex (left) and right precuneus (right) volumes negatively correlated with the number of boxes opened in the IST. The data are shown for healthy volunteers (HV) and binge drinkers (BD)

In the IST (Clark *et al*. 2006), participants were presented with a 5 × 5 array of grey boxes on a touch‐screen monitor (Fig. 2). Upon being touched, each box opened to reveal one of two colours, shown as panels below the matrix. The objective was to decide which of the two colours was predominant in the matrix, for a particular trial, by opening a sufficient number of boxes in order to be able to make that decision. Two conditions were measured (10 trials each): a ‘fixed win’ condition in which the participant was awarded 100 points for a correct decision regardless of the number of boxes opened and a ‘decreasing win’ condition (cost‐per‐sample condition) in which the number of points that could be won for a correct decision started at 250 and decreased by 10 points for every box touched. The primary outcome measure was the number of boxes opened. Total points, numbers of errors and P (correct), which is a measure that quantifies the extent of the information revealed on a trial‐by‐trial basis (Clark *et al*. 2006), were also assessed. IST data from 30 subjects were used as covariate in the imaging analysis.

### Delay discounting task (DDT)

Delay discounting was measured using the Monetary Choice Questionnaire (Kirby & Marakovic 1996), composed by 27 items, in which participants choose between a small immediate reward and a larger delayed reward. The primary outcome measure was the discount parameter K.

### Statistical analysis of behavioural outcomes

The subject characteristics, impulsivity measures and primary outcomes were assessed using independent *t*‐tests. The data were inspected for outliers [> 3 standard deviation (SD) from the group mean], which were removed from analysis. The behavioural outcomes were assessed for normality (Shapiro–Wilks *P* < 0.05 and histogram inspection). The Beads and the IST data were square root transformed. As the DDT data were right skewed, a log10 transformation was applied. The secondary outcomes of the IST were assessed using mixed‐measures ANOVA to assess the role of cost on sampling error, total points and Pcorrect with group as a between‐subjects factor and cost as a within‐subjects factor.

### Computational modelling analysis of probabilistic reasoning in the beads task

We further analysed the beads task using a Bayesian decision‐making model as previously reported by Moutoussis *et al*. (2011). This model assumes that given quantifiable goals motivating a person, and a model of the environment that they use to predict outcomes following actions, optimal decisions follow the ‘ideal Bayesian observer’. The Bayesian model contains two key parameters to explain precipitous or inconsistent decisions (Moutoussis *et al*. 2011). The first is the subjective cost for each piece of information sampled. This can be explicitly defined by the experimenter consistent with the ‘decreasing win’ or cost condition of the IST in which a penalty is incurred for each piece of evidence gathered. The subjective cost can also be participant related, e.g. to intolerance of uncertainty or delay discounting over the brief period that the task takes to complete. The other key parameter is a ‘cognitive noise’, which determines both choice consistency and the extent to which cognitively distant outcomes can influence current choices. The ‘cognitive noise’ parameter thus provides a measure of how much decision making is influenced by the more cognitively difficult process of ‘thinking ahead’.

We estimated the cost‐of‐sampling (Cs) and cognitive noise (T) parameters for each participant using expectation maximization, using the initial assumption that the parameter values for all participants came from gamma distributions over Cs and T. This model is analogous to the decision threshold models used in much of probabilistic decision literature. However, it allows participants to optimally relax their decision boundaries as the end of the task approaches if it is unlikely that good evidence will accumulate. Previous research suggests that this Bayesian model gives a better account of healthy people's behaviour. Further details of the fitting procedure and comparison with the fixed‐decision‐threshold models can be obtained from Moutoussis *et al*. (2011).

### Image data acquisition

Imaging data were acquired using a Siemens 3T Tim Trio scanner (Siemens Medical System Systems, Erlangen, Germany) with a 32‐channel head coil at the Wolfson Brain Imaging Center at the University of Cambridge. Scans were obtained using a T1‐weighted structural image with the following parameters: repetition time = 2300 ms; echo time = 2.98 ms; matrix: 240 × 256 × 176 mm, voxel size 1 × 1 × 1 mm.

### Image processing and analysis

The three‐dimensional T1‐weighted images were pre‐processed with Statistical Parametric Mapping software (SPM8) (http://www.fil.ion.ucl.ac.uk/spm). The images were reoriented, aligning the origin approximately to the anterior commissure. Then, the images were segmented into different tissue classes using New Segment, which employs tissue probability maps to assign a probability of each voxel belonging to a particular tissue type (cerebrospinal fluid, white matter and grey matter). The volume of these tissues was then summed to provide an estimate of the total intracranial volume for each participant. DARTEL (Ashburner 2007), a diffeomorphic method, was used to generate an average template data to which the data are iteratively aligned for the non‐linear deformation of the grey and white matter images. To transform these template‐space images into ICBM152 MNI space (http://www.bic.mni.mcgill.ca/ServicesAtlases/ICBM152NLin2009), the DARTEL template was registered to MNI space using an affine transformation allowing the transformations to be combined so that the individually spatially normalized scans are in MNI space. All images were smoothed using a 10 mm full width at half maximum isotropic Gaussian kernel in the final normalization step.

The analyses were corrected for the total intracranial volume using proportional scaling and an explicit mask created using the binarized image from the SPM brain mask template using ImCalc. Grey matter volumes were analysed using separate covariate analyses for transformed outcomes of the number of beads of the JTC task, number of boxes opened for the IST task and K‐value of the DDT as independent variables. Age and gender were included as covariates of no interest; and in a separate analysis, the AUDIT score was also included as a covariate of no interest. Whole brain voxel‐wise group comparisons were performed using a cluster extent threshold correction. The cluster extent threshold correction was calculated at 19 voxels at *P* < 0.001 whole‐brain uncorrected, which corrected for multiple comparisons at *P* < 0.05 assuming an individual‐voxel type I error of *P* = 0.01 (Slotnick *et al*. 2003).

## Results

Thirty BD were compared with 30 age‐ and gender‐matched HV. Alcohol intake per week in units was: HV: 4.78 (2.41); BD: 13.20 (SD 4.85), *t* = −8.16, *P* < 0.0001). BD had higher AUDIT scores and were more impulsive on the UPPS and specifically on the subscales of positive and negative urgency (Table 1).

**Table 1:** Participant characteristics: binge drinkers (BD) and healthy volunteers (HV)

	HV	BD	t (*P*)
*N*	30	30	
Males/Females	17/13	17/13	
Age	21.85 (3.26)	22.22 (3.35)	−0.40 (0.69)
IQ	116.72 (6.19)	116.61 (5.26)	−0.44 (0.67)
AUDIT	4.00 (2.76)	15.48 (5.45)	−8.564 (< 0.0001)
BDI	4.73 (4.82)	7.14 (5.71)	−0.842 (0.403)
STAI	36.13 (11.01)	41.93 (12.12)	−1.117 (0.269)
Negative urgency	25.08 (6.21)	30.16 (6.41)	−3.287 (0.002)
Premeditation	21.59 (6.04)	24.63 (6.52)	−1.979 (0.052)
Perseveration	20.03 (3.73)	21.43 (4.35)	−1.423 (0.160)
Sensation seeking	34.54 (7.67)	36.03 (7.44)	−0.803 (0.425)
Positive urgency	24.05 (7.67)	29.13 (8.94)	−2.502 (0.015)

Data are presented as mean (standard deviation) and one‐tailed.

AUDIT = Alcohol Use Disorders Identification Test; BDI = Beck Depression Inventory; IQ = interquartile; STAI = Spielberger State and Trait Anxiety Inventory.

### Behavioural results

**Figure 3:**
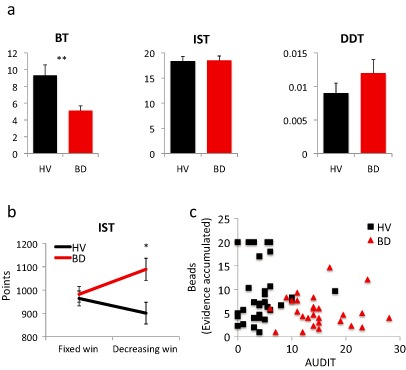
Behavioural results. (a) Group comparisons of binge drinkers (BD) and healthy volunteers (HV) for the number of beads drawn on the beads task (BT) and the number of boxes opened on Information Sampling Task (IST) and K‐value of the Delay Discounting Task (DDT). For illustration purposes, the raw data are shown rather than the transformed data, **P < 0.005. (b) Total points won for the IST comparing BD with HV, *P < 0.05. Group and group‐by‐cost interaction. (c) Correlation analyses between samples drawn in the BT and the Alcohol Use Disorders Identification Test (AUDIT) scores for each group (BD and HV)

One beads and two DDT score outliers in the BD group were removed from the data set. In the primary outcome measures, BD subjects selected fewer beads compared with HV in the beads task (*t* = 3.148, d.f. = 57, *P* = 0.003) (Fig. 3) . The confidence ratings were not significantly different [HV: 329.54 (SD 89.41); BD: 322.36 (SD 69.00), *t* = 0.344, d.f. = 57, *P* = 0.732]. The groups were not significantly different in the number of boxes opened on the IST (IST boxes) in the ‘fixed win’ condition (*t* = −0.209, d.f. = 58, *P* = 0.835) or in the delay discounting K (*t* = 1.173, d.f. = 56, *P* = 0.245).

In the secondary analyses of the IST outcome measures, there was a main effect of group in the number of total points (*F*(1,58) = 5.281, *P* = 0.025) and a group‐by‐cost interaction (*F*(1,58) = 5.192, *P* = 0.026). There was no main effect of cost (*F*(1,58) = 0.349, *P* = 0.557). BD subjects had greater overall number of total points (main group effect) driven by an increase in total points in the cost‐per‐sample condition. One would expect that all the participants would make more points in the ‘fixed win’ condition, where there is no cost associated. This was observed by the HV group. However, BD subjects overall scored more points (main group effect) driven by a significant increase in total points in cost‐per‐sample compared with the ‘fixed win’ condition (group‐by‐cost interaction). There was a main effect of cost in the sampling error rate (*F*(1,58) = 43.101, *P* < 0.0001) but no group effect (*P* = 0.283) or group‐by‐cost interaction (*P* = 0.755). There was a main effect of cost in Pcorrect (*F*(1,58) = 99.465, *P* < 0.0001) but no group effect (*P* = 0.505) or group‐by‐cost interaction (*P* = 0.449).

To further assess the measure to ensure adequate sample size, we compared BD subjects with twice the number of HV [*n* = 60; males = 34; age 21.95 (SD 3.88) (*t* = 0.325, *P* = 0.746)] [reported in log10 K‐value: HV 0.005 (SD 0.005), BD 0.005 (SD 0.005), d.f. = 88, *t* = −0.348, *P* = 0.729].

We then assessed the relationship with binge drinking severity using a Pearson's correlation with the AUDIT score. There was a significant negative correlation between the AUDIT score and samples drawn in the beads task across groups (i.e. greater impulsivity was correlated with greater alcohol severity) (Pearson's correlation coefficient = −0.315, *P* = 0.030) but not with the IST boxes (−0.024, *P* = 0.843) or with temporal discounting K (0.149, *P* = 0.218). The groups were analysed separately to assess the impact of the AUDIT score on the beads task: neither group demonstrated a significant correlation (*P* > 0.05) (Fig. 3).

There were no significant relationships between the beads drawn in the beads task with IST boxes opened (0.168, *P* = 0.248) or K (−0.027, *P* = 0.847). In the secondary outcome measures, there was a significant negative correlation across groups between the number of beads drawn in the beads task with the sampling error (−0.271, *P* = 0.035) and total points (0.282, *P* = 0.028) in the ‘fixed win’ condition of the IST but not in the cost‐per‐sample condition (−0.178, *P* = 0.169; 0.029, *P* = 0.827, respectively).

For the modified beads task with reward feedback, 29 subjects (14 BD and 15 HV) were tested after the MRI. The same BD subject excluded in the previous data set was also excluded in this current data set. There was a trend towards a group difference [beads opened: BD: 5.21 (SD 3.74); HV 8.07 (SD 4.10), *t* = 26, d.f. = 1.96, *P* = 0.067]. We assessed the relationship between the beads task without outcome and the beads task with reward feedback using Pearson's correlation to assess test‐retest effects. The two versions of beads task were significantly correlated (r = 0.605, *P* = 0.003).

There were no correlations between the UPPS subscores and IST or JTC (all *P* > 0.05).

### Computational modelling results

Analysis including all participants showed that HV had a lower ‘cognitive noise’ than BD. However, the HV group included more participants deciding at the maximum draws (20 draws). These participants may not explicitly take cognitively distant outcomes into account but use a simple heuristic instead. We therefore conservatively analysed the data excluding the ‘20 draws’ participants (for details, see Supporting Information Appendix S1), leaving N_control_ = 24 and N_binge_ = 28.

We obtained a good model fit with median log probability per draw about −2.0, attesting to the validity of the model. Both the fitted mean and the SD of the cost‐per‐sample, Cs, were negligible compared with the noise parameter for both HV and BD groups (means ∼ 0.02, SDs ∼ 0.01). This is evidence against subjective cost considerations causing the differences in draws to decision.

**Figure 4:**
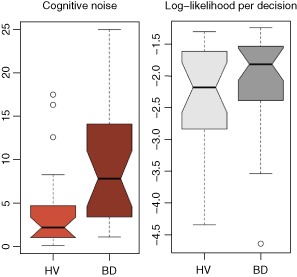
Computational modelling results. Binge drinkers (BD) showed higher ‘cognitive noise’ than healthy volunteers (HV) and greater variability within the group. The plots show medians, interquartiles (solid), full ranges (whiskers) and outliers (circles). The notches are non‐parametric confidence intervals

On the other hand, the groups differed significantly with respect to the noise parameters, as shown in Fig. 4. The median noise parameter for the healthy group was lower (Wilcoxon *P* = 0.02) and the variability within the BD group was greater.

### Imaging results

VBM analysis was conducted investigating the relationship between the impulsivity measures and brain volume with age and gender as covariates of no interest and also with and without AUDIT score as a covariate of no interest. The number of beads drawn in the beads task (lower impulsivity) was positively correlated with greater volume in the left (L) cerebellum (−39 −84 −21, cluster size = 217, Z = 4.15), L DLPFC (−22 45 28, cluster size = 271, Z = 3.87), L inferior parietal cortex (−45 −44 30, cluster size = 98, Z = 3.75) and right (R) thalamus (17 −26 6, cluster size = 50, Z = 3.28) (Fig. 1). On an exploratory basis, we analysed BD and HV separately focusing on the L DLPFC (HV: *R* ^2^ = 0.43, *P* = 0.007; *R* ^2^ = 0.76, *P* < 0.0001) and L parietal cortex (HV: *R* ^2^ = 0.48, *P* = 0.005; BD: *R* ^2^ = 0.60, *P* = 0.001). In order to further explore the relationship between beads drawn and the neuroanatomical correlations in the BD and HV group, we also conducted a *t*‐test comparing BD and HV with age and gender as covariates of no interest focusing on the regions identified in the correlation analysis. There were no significant group differences between BD and HV in the cerebellum, DLPFC, inferior parietal or thalamus. With the addition of AUDIT along with age and gender as covariates of no interest, the following regions remained significant at a whole brain family‐wise error cluster corrected level: L DLPFC (cluster size = 202, Z = 3.93), L cerebellum (cluster size = 150, Z = 3.88), L inferior parietal (cluster size = 52, Z = 3.49), R thalamus (cluster size = 21, Z = 3.19). There were no negative correlations or interaction effects.

For the IST (greater number of boxes opened or lower impulsivity), there was a negative correlation with L dorsal cingulate (−17 36 18, cluster size = 283, Z = 4.45) and R precuneus (8 −57 60, cluster size = 157, Z = 3.77) (Fig. 1). With the addition of AUDIT as a covariate, the same regions remained significant (L dorsal cingulate: cluster size = 268, Z = 4.38; R precuneus: cluster size = 158, Z = 3.74). There were no positive correlations or interactions. There were no whole brain cluster level corrected significant findings for the log10 DDT measure.

## Discussion

We show using both behavioural analysis and computational modelling that binge drinkers accumulated less evidence prior to a decision in the beads task. This enhanced reflection impulsivity in the beads task correlated with alcohol severity as measured using the AUDIT scores; however, as neither group demonstrated a separate correlation, this finding may be specific to group differences rather than a function of alcohol use severity. Although we did not find differences in evidence accumulated in the IST, in response to an explicit cost to the evidence accumulated, binge drinkers improved the capacity to integrate information and increased the number of total points earned. These findings dovetail with our previous finding that binge drinkers were more risk‐taking in the anticipation of unlikely losses which improved with the exposure to the explicit experience of the probability and loss outcome (Worbe *et al*. 2014). We did not find any differences in delay discounting despite further analyses increasing the sample size of healthy volunteers.

### Beads task and information sampling task: task comparisons

The dissociation in the IST and beads task results was unexpected given that the tasks test similar concepts. However, a similar dissociation has been shown in the psychosis literature. The beads task is consistently impaired in studies in schizophrenia (Fine *et al*. 2007; Moutoussis *et al*. 2011) but in a recent study there were no differences between first‐episode psychosis patients and healthy volunteers on the IST (Huddy *et al*. 2013). The disparity is likely a function of task differences. The IST presents information in a very explicit manner. First, the use of a 5 × 5 grid in the IST shows the total amount of information available to be sampled as a constant reminder, possibly acting as an explicit external relative anchor and encouraging ‘thinking ahead’ of all possible options and overall representation of the task. In contrast, in the beads task, although subjects are explicitly told that they can sample up to 20 beads in the instruction phase, this information is less visually explicit. Subjects must rely on their own possibly less constant internal anchor, if at all, and may be less likely to always consider all possible options and thus be more sensitive to impulsive decisions. This feature may be extremely relevant for explaining our dissociated findings between the IST and the beads task, considering that BD are less influenced by internal representation of future outcomes (as shown by the computational analysis). Although the IST may be more transparent and reduce uncertainty of the end point or total available information, we suggest that the beads task may be more ecologically valid as the total information available is not always an explicitly known entity.

Second, the evidence is sampled from differing known probabilities. In the beads task, bead sequences are generated from jars of known probabilities whereas in the IST, the generative probability distribution from which the coloured boxes are sampled is unknown. Furthermore, it may soon become apparent to participants that this generative probability is close to 50:50, pushing them towards caution. Thus, in the beads task, the easier probability structure but more vague task structure may increase sensitivity to impulsive decisions.

Third, binge drinking has been associated with impairments in working memory (Stephens & Duka 2008; George *et al*. 2012). However, both tasks visually display the amount of information acquired to control for working memory (Menon *et al*. 2006) although in different formats. Fourth, differences in monetary rewards are unlikely to explain different task results. In the ‘fixed win’ condition, the IST is associated with winning points if correct while in our first version of the beads task, the task was not associated with an explicit reward. However, our second version of the beads task was associated with a reward outcome if correct and was strongly correlated with the first uncosted task.

The IST allows for an explicit introduction of cost to the evidence sampled. Here, there was an effect in binge drinkers on improving total points accumulated despite the same available information suggesting that an explicit cost may press binge drinkers to be more cautious in integrating the information gathered. The introduction of an explicit cost to evidence sampled or loss feedback to risk evaluation may shift binge drinkers towards a more cautious approach with more optimal information integration to maximize outcomes and towards greater risk aversion (Worbe *et al*. 2014).

### Bayesian modelling

The computational analysis gave further insight into the behavioural results. The Bayesian probabilistic reasoning model gave a good account of decision making for most participants in the beads task, although a few participants may have followed heuristics not included in our model. In contrast, individuals with psychosis characterized along similar lines (Moutoussis *et al*. 2011; Huddy *et al*. 2013) have been found to be ‘worse Bayesians’ than healthy volunteers. The Bayesian model of decision making showed that the earlier decisions of the binge‐drinking group were related to being less influenced by future outcomes or ‘thinking ahead’ which is cognitively more demanding. There was no evidence for early decisions being accounted by attaching a greater implicit cost to gathering more information, such as more rapid decisions during the beads task related to delay discounting. This was congruent with our lack of group difference in the DDT.

### Relationship with brain volume

We further compared the volumetric correlates of the beads task and IST across subjects irrespective of the experimental group. Greater impulsivity as indexed by lower evidence accumulation in the beads task was associated with smaller L DLPFC and L inferior parietal volumes. In contrast, greater impulsivity as indexed by lower evidence accumulation in the IST was associated with greater L dorsal cingulate and R precuneus volumes.

The mechanisms underlying evidence accumulation in these tasks can be subdivided into the stage of decision making such as during the evidence seeking or the decision process. A previous fMRI study of the beads task has shown parietal activity during the evidence‐seeking process and DLPFC activity during both evidence‐seeking and the final‐decision phase (Furl & Averbeck 2011). Similarly, in another fMRI study investigating evidence accumulation, greater uncertainty during evidence accumulation was associated with dorsal anterior cingulate cortex (ACC) and precuneus activity whereas greater uncertainty during decision execution was associated with greater lateral frontal and parietal activity (Stern *et al*. 2010).

In addition to underlying error and conflict monitoring processes (Scheffers & Coles 2000; Botvinick *et al*. 2001), dorsal ACC has also been implicated in coding unexpected or unpredicted outcomes during evidence accumulation, e.g. integrating a draw that was inconsistent with expectations established by previous observation of draws (Oliveira, McDonald & Goodman 2007; Stern *et al*. 2010). The parietal cortex has been suggested to play several roles including signalling the final decision and confidence (Kiani & Shadlen 2009; Stern *et al*. 2010) and also during evidence accumulation, by comparing the costs of seeking additional evidence to the potential gains and losses of an immediate reward‐related decision. The DLPFC is important in the resolution of uncertainty when subjects deal with limited knowledge (Huettel *et al*. 2005) and in the integration of cost and benefits (Basten *et al*. 2010). The DLPFC is also implicated in working memory (Balconi 2013) although this was controlled in the task design. Insular activity has also been shown to be modulated by the action values or expected reward in a previous fMRI study that assessed evidence accumulation using the beads task (Furl & Averbeck 2011). However, our study focuses on volumetric differences and did not show any correlation with insular volumes. Overall, these volumetric findings might suggest differences between the beads task and IST.

### Delay discounting

We did not show differences between binge and non‐binge drinkers on the measure of delay discounting despite increasing the sample size of HV. Delay discounting has been consistently observed in AUD individuals (Bickel *et al*. 2014). In binge drinkers, the findings for this measure are less clear. A study that assessed adolescents with greater alcohol consumption using a two‐choice real‐time DDT found enhanced delay discounting (Moreno *et al*. 2012). Another study that assessed a group specifically defined as binge drinkers also found elevated delay discounting in young adolescents (age 14) using the Monetary Choice Questionnaire, a result which was not predictive of future binge drinkers (Whelan *et al*. 2014). However, similar to Sanchez‐Roige *et al*.'s (2014a) study, we did not find differences in delay discounting in young adults using the Monetary Choice Questionnaire. We suggest that the inconsistent findings in delay discounting in binge drinkers may be related to differences as a function of age (adolescence versus young adults), group definition (higher alcohol consumption versus specifically defined binge drinkers) or tasks (real‐time reward versus more hypothetical long‐term reward). Further studies are indicated to clarify these differences.

### Limitations

One limitation of this study was the use of two versions of the beads task. However, previous studies have demonstrated good test‐retest reliability with the beads task (Peters & Garety 2006; Woodward *et al*. 2009), and furthermore, we show a positive correlation between the two results and a trend towards a difference between groups. Only 14 BD and 15 HV completed the second version of the beads task for the volumetric analysis. However, with respect to the analysis of the relationship between this measure and brain volume (and not of group differences), the sample size of 29 was appropriate.

## Conclusion

Our findings suggest differences between the beads task and IST implicating lateral cortical regions and mesial cortical regions, respectively. Our study provides evidence for impairments in reflection impulsivity but not delay discounting in binge drinking. The beads task has been shown to be sensitive to group differences in substance and behavioural addictions and we similarly confirm these findings in a binge‐drinking group. We further emphasize the role of explicit cost‐per‐sample in improving optimal information integration consistent with previous findings of explicit information on probability and loss in decreasing risk‐taking behaviours (Worbe *et al*. 2014). We conclude that binge drinkers may have a relatively specific deficit in deciding on the basis of future outcomes that are more difficult to represent.

## Supporting information


**Appendix S1** Methods and results.
