# Supplementary material for: Reflection impulsivity in binge drinking: behavioural and volumetric correlates
Source: Addict Biol. 2015 Feb 11;21(2):504–15. doi: 10.1111/adb.12227 (PMC4766871; doi:10.1111/adb.12227)
Supplement: Supplementary file 1 — Appendix S1 Methods and results. [file ADB-21-504-s001.docx]

**Supporting information**

**Methods**

*Delay discounting task*

The primary outcome measure is a hyperbolic discount parameter (k) that gives an estimate of the rate of delayed discounting and can be calculated as follows: V = A/(1+kD), where V is the present value of delayed reward A and D is the length of the delay. The higher the k value the greater the discounting and the impulsive choice. The k value of small, medium and large magnitude choices were averaged for the final k value.

**Results**

*Computational modelling results*

Computational modelling preliminary analyses showed that the overall sample was inhomogeneous. Both the control and the BD group each contained a larger subgroup conforming to the model described in the methods section, and a smaller distinct subgroup that took all 20 draws to decide for at least two of the three trials. The model can account for these participants by postulating a very low cognitive noise. However, it is likely that the "20 draws" subgroup (Control: N=6; BD: N=2) used a different heuristic than the others; for instance, they may have simply decided to use the maximum information and stuck to it.
